# miR‐181c‐5p mediates simulated microgravity‐induced impaired osteoblast proliferation by promoting cell cycle arrested in the G_2_ phase

**DOI:** 10.1111/jcmm.14220

**Published:** 2019-02-14

**Authors:** Zhongyang Sun, Ying Li, Han Wang, Min Cai, Shanshan Gao, Jing Liu, Liangcheng Tong, Zebing Hu, Yixuan Wang, Ke Wang, Lijun Zhang, Xinsheng Cao, Shu Zhang, Fei Shi, Jianning Zhao

**Affiliations:** ^1^ Department of Orthopedics, Affiliated Jinling Hospital Medical School of Nanjing University Nanjing China; ^2^ Department of Orthopedics, Junxie Hospital Anhui Medical University Nanjing China; ^3^ The Key Laboratory of Aerospace Medicine, Chinese Ministry of Education Fourth Military Medical University Xi'an China; ^4^ Department of Orthopedics, Affiliated Hospital of Air Force Aviation Medicine Research Institute Fourth Military Medical University Beijing China; ^5^ Medical Services Section, Junxie Hospital Anhui Medical University Nanjing China; ^6^ Department of Pharmacy, Junxie Hospital Anhui Medical University Nanjing China

**Keywords:** cell cycle, cell proliferation, cyclin B1, miR‐181c‐5p, osteoblast, simulated microgravity

## Abstract

Impaired osteoblast proliferation plays fundamental roles in microgravity‐induced bone loss, and cell cycle imbalance may result in abnormal osteoblast proliferation. However, whether microgravity exerts an influence on the cell cycle in osteoblasts or what mechanisms may underlie such an effect remains to be fully elucidated. Herein, we confirmed that simulated microgravity inhibits osteoblast proliferation. Then, we investigated the effect of mechanical unloading on the osteoblast cell cycle and found that simulated microgravity arrested the osteoblast cell cycle in the G_2_ phase. In addition, our data showed that cell cycle arrest in osteoblasts from simulated microgravity was mainly because of decreased cyclin B1 expression. Furthermore, miR‐181c‐5p directly inhibited cyclin B1 protein translation by binding to a target site in the 3′UTR. Lastly, we demonstrated that inhibition of miR‐181c‐5p partially counteracted cell cycle arrest and decreased the osteoblast proliferation induced by simulated microgravity. In conclusion, our study demonstrates that simulated microgravity inhibits cell proliferation and induces cell cycle arrest in the G_2_ phase in primary mouse osteoblasts partially through the miR‐181c‐5p/cyclin B1 pathway. This work may provide a novel mechanism of microgravity‐induced detrimental effects on osteoblasts and offer a new avenue to further investigate bone loss induced by mechanical unloading.

## 1 INTRODUCTION

Bone is a dynamically remodelled tissue that requires gravity‐mediated mechanical stimulation for maintenance of the mineral content and structure.^1^, ^2^ Numerous studies have shown that mechanical stimulation increases bone formation in modelling the skeleton, whereas reduced mechanical loading, as observed in patients subjected to prolonged immobilization or bed rest and in astronauts in a microgravity environment, results in reduced bone mass.^3^, ^4^, ^5^, ^6^ Impaired osteoblast proliferation is believed to play an important role in microgravity‐induced bone loss.^7^, ^8^ The mechanisms by which microgravity exerts these detrimental effects on osteoblast proliferation remain unclear and merit further research.

Accumulating evidence has indicated that an imbalance of cell cycle progression may result in abnormal cell proliferation and even carcinogenesis.^9^, ^10^, ^11^ Cell cycle arrest induced by microgravity has been observed in many cell types, including myoblasts,^12^ Arabidopsis cells,^13^ leucocytes^14^ and bone marrow mesenchymal stem cells.^15^, ^16^ Although microgravity has been implicated in halting cell cycle progression, the precise mechanisms behind this phenomenon are not yet fully understood. Cell cycle progression is critically regulated by the sequential activation of cyclins and cyclin‐dependent kinases (Cdks).^17^, ^18^ In mammalian cells, the transition from the G_2_ phase into mitosis is controlled by the activation of the maturation promoting factor, the major component of which is the cyclin B1‐Cdc2 kinase complex.^17^, ^18^, ^19^ The Cdc2 (also known as Cdk1) catalytic subunit is regulated by a series of coordinated phosphorylation and dephosphorylation events.^17^ Activation of Cdc2 is prevented by its phosphorylation at Thr14/Tyr15 by the protein kinases Wee1 and Myt1.^17^, ^20^, ^21^ Dephosphorylation of Thr14/Tyr15 by the protein phosphatase Cdc25 eventually activates the Cdc2‐cyclin B1 complex, which allows for progression to mitosis.^17^, ^22^ Cdc2 activity is also regulated by the availability of the cyclin subunits. During the S phase, cyclin B1 mRNA and protein begin to accumulate, and their levels are highest during the G_2_/M phase.^17^ As cells progress through mitosis, cyclin B1 is ubiquitinated and degraded by the anaphase‐promoting complex.^17^, ^23^ In addition, the activated cyclin B1‐Cdc2 kinase complex translocates from the cytoplasm into the nucleus, which is required for cells to enter mitosis.^17^

Recent studies have shown that in addition to the ubiquitination and anaphase‐promoting complex mentioned above, there are other factors that participate in cyclin B1 regulation.^24^, ^25^, ^26^ MicroRNA (miRNA), which is a family of small, single‐stranded non‐coding RNA molecules, has been well studied and it functions in the silencing and post‐transcriptional regulation of gene expression.^27^, ^28^ Gene expression is repressed by miRNAs through base‐pairing with complementary sequences within mRNA molecules.^29^, ^30^ It is estimated that miRNAs regulate more than 30% of human protein‐coding genes, demonstrating the essential role of miRNAs in modulating gene expression.^31^, ^32^ Several studies have shown that miR‐199a‐5p,^33^ miR‐410^34^ and miR‐379^35^ mediate the cell cycle by directly regulating cyclin B1 (Ccnb1) expression in several cell types of cells. However, the function of these miRNAs in osteoblasts has not been confirmed.

Taken together, these data suggest that impaired osteoblast proliferation plays an important role in microgravity induced‐bone loss and that cell cycle disorders may lead to decreased osteoblast proliferation. In addition, miRNAs may take part in the regulation of the cell cycle in osteoblasts. However, little is known about whether microgravity exerts an influence on the cell cycle in osteoblasts, and the possible mechanisms underlying this effect remain unclear. In the present study, we have been suggested that simulated microgravity inhibits cell proliferation and induces cell cycle arrest in primary mouse osteoblasts through a miRNA/cyclin pathway. We therefore examined the effects of simulated microgravity on osteoblast proliferation and cell cycle progression with methods including 5‐ethynyl‐2′‐deoxyuridine (EdU) labelling, immunostaining approaches and flow cytometry. Then, we used real‐time quantitative PCR (qPCR) and specific immunostaining approaches to examine the effects of simulated microgravity on the function of the cyclin B1‐Cdc2 kinase complex. In addition, we assessed the role of cyclin B1 and miR‐181c‐5p in mediating cell proliferation and cell cycle progression in primary mouse osteoblasts under simulated microgravity conditions. This study may provide a novel mechanism for bone loss caused by mechanical unloading.

## 2 MATERIALS AND METHODS

### 2.1 Cell culture and transfection

Primary mouse osteoblasts were isolated as described previously.^36^, ^37^ Cells were used at passages 3‐5. Then, cells were confirmed to be osteoblasts using a phenotype characterized by Runx2 and ALP expression and the capacity to form mineralized bone nodules (data not shown). To better observe the effects of simulated microgravity, cells were cultivated for 24 hours with serum‐free medium to implement cell cycle synchronization and serum starvation induced growth arrest in the G_1_ phase. In addition, nocodazole (RiboBio, China) is frequently used to synchronize the cell cycle. Cells treated with nocodazole arrest at mitosis, and this treatment served as a positive control. 2T3 cells were subjected to luciferase assay after transfection for further analysis.

For transfection of plasmids or miRNA regulators, Lipofectamine2000 (Invitrogen, USA) was used according to the manufacturer′s instruction. pcDNA3.1‐cyclin B1 or pcDNA3.1 empty vector (Berke, China) was transfected at a concentration of 200 ng/μL, and inhibitor‐miR‐181c‐5p or inhibitor negative control (NC) (RiboBio) was transfected at the concentration of 100 nmol/L. The sequences of miR‐181c‐5p (Accession mimat0000674) inhibitor and the negative control are listed as follow: miR‐181c‐5p inhibitor, 5′‐UUG UAA GUU GGA CAG CCA CUCA‐3′, and the negative control, 5′‐GCA GUA CGC CCC AGG CGC UUU‐3′. Normal or transfected cells were then subjected to clinorotation for 48 hours and harvested for further analysis.

### 2.2 Clinorotation to simulate microgravity

Because of the limitation of spaceflight missions, most studies on the biological effects of microgravity are conducted using ground‐based analogs. The clinostat (Astronaut Research and Training Center, China) is an effective tool to simulate microgravity. Cells were exposed to clinorotation for 48 hours at 24 r.p.m. Cells grew for 24 hours and adhered to the coverslips which were inserted into the fixture of the chambers. The chambers were divided into two groups: horizontal rotation control and clinorotation.^36^, ^38^, ^39^ To confirm the microgravity achieved by this clinostat, we tested the effects of the simulated microgravity on biological characteristics of primary mouse osteoblasts in this experimental condition (Figure S1).

### 2.3 EdU labelling assay

EdU labelling was performed according to the manual of EdU labeling kit (RiboBio). Cells were visualized under an inverted microscope linked to a confocal scanning unit (FluoView 1000; Olympus, Japan). The EdU‐positive cells (green) were counted using Image‐Pro Plus 6.0 software (Media Cybernetics, USA).^40^, ^41^

### 2.4 Cell counting kit‐8 assay

Cell proliferation was evaluated by WST‐8 (4‐{3‐(2‐methoxy‐4‐nitrophenyl)‐2‐(4‐nitrophenyl)‐^2^H‐5‐tetrazolio}‐1,3‐benzene disulfonate sodium salt; Cell Counting Kit‐8) assay. In brief, WST‐8 solution (Dojindo, Japan) was added to the culture medium and cells were incubated at 37°C for an additional 3.5 hours. The absorbance of reaction solution at 450 nm was measured using a microplate reader (Thermo Scientific, USA).^40^, ^42^

### 2.5 Western blot analysis

The cells were lysed in radio immunoprecipitation assay (RIPA) buffer (Thermo Fisher Scientific, USA) containing a protease inhibitor cocktail (Roche, Switzerland). The resulting bands were quantified through densitometry with the ImageJ software using the proliferating cell nuclear antigen (PCNA) antibody (1:3000, ab29; Abcam, USA), the histone H_3_ antibody (1:5000, ab8580; Abcam), the histone H_3_ (phospho Ser10) antibody (1:1000, ab5176; Abcam), the Cdc2 antibody (1:5000, ab18; Abcam), the Cdc2 (phospho Tyr15) antibody (1:1000, ab47594; Abcam), the cyclin B1 antibody (1:10 000, ab32053; Abcam) and GAPDH antibody (1:5000, ab8245; Abcam).^40^

### 2.6 Cell cycle assay

Cells in different groups were trypsinized (HyClone, USA), washed once with PBS and fixed with 70% ethanol overnight at 4°C. After fixation, cells were washed once with PBS and resuspended in PBS/0.1% Triton X‐100 and incubated with 50 U DNase‐free RNaseA (Calbiochem, Germany) (30 minutes, room temperature). After incubation, cells were stained with propidium iodide (20 mg/mL in PBS, 15 minutes at room temperature). Flow cytometry (FCM) analysis was performed with a flow cytometer (BD Biosciences, USA).^18^

### 2.7 Immunofluorescence staining assay

Cells from each group were fixed in 4% (vol/vol) paraformaldehyde for 30 minutes. Cells were incubated overnight at 4°C with oscillation with primary antibody against either histone H_3_ (phospho Ser10) (1:300, ab5176; Abcam), Cdc2 (1:300, ab18; Abcam), Cdc2 (phospho Tyr15) (1:200, ab18; Abcam) or cyclin B1 (1:100, ab32053; Abcam). Then, cells were incubated in the dark for 1 hour at room temperature using Alexa Fluor 488 conjugated secondary antibody (1:200; Invitrogen, USA). Cells were counterstained for 10 minutes in the dark with the nuclear dye Hoechst (Hoechst AG, Germany) diluted 1:4000 in PBS. The fluorescence was captured and observed using an inverted microscope linked to a laser scanning confocal microscope (FluoView1000; Olympus).^18^, ^38^

### 2.8 Immunoprecipitation and Cdc2‐cyclin B1 kinase assay

Cell lysates obtained from each group were prepared as described previously.^17^, ^43^ Lysate supernatants were incubated with an anti‐cyclin B1 antibody (1:50, ab32053; Abcam) using a Catch and Release (version 2.0) reversible immunoprecipitation system (Merck Millipore, Germany). Equal amounts of Cdc2‐cyclin B complex from each sample were washed three times with 1 mL of modified RIPA buffer. Then, the Cdc2‐cyclin B1 kinase activity in cells was measured using a Cdc2‐cyclin B kinase assay kit (Cyclex Nagano, Japan) according to the manufacturer's protocol.^17^

### 2.9 mRNA and miRNA expression assays

For mRNA, cDNA was synthesized using the Primerscript RT Kit (TaKaRa, Japan). The amplification was performed at 95°C for 45 seconds, followed by 40 cycles of 58°C for 45 seconds, 72°C for 60 seconds. The primers pairs were as follows: Ccnb1 (GenBank accession NM_172301.3): F‐5′‐GGA ACG GCT GTT AGT GTT TAG C‐3′ and R‐5′‐AAA GCT TTC CAC CAA TAA ATT TTA TT‐3′; GAPDH (GenBank accession NM_008084): F‐5′‐CAT GTT CCA GTA TGA CTC CAC TC‐3′ and R‐5′‐GGC CTC ACC CCA TTT GAT GT‐3′.

For miRNA, cDNA was synthesized using the miRNA First Strand Synthesis kit (Agilent Technologies, USA). The universal reverse primer: 5′‐TGG TGT CGT GGA GTC G‐3′, miR‐181c‐5p (accession mimat0000674) specific forward primer: 5′‐UUG UAA GUU GGA CAG CCA CUC A‐3′. Amplification was carried out at 95°C for 2 minutes, followed by 40 cycles of 95°C for 10 seconds, 60°C for 40 seconds.

Quantification of gene expression was performed with the comparative threshold cycle (ΔΔC_T_) method. GAPDH was used as a control for Ccnb1 mRNA quantification and small nuclear RNA U6 was used as a control for miR‐181c‐5p.^38^, ^40^

### 2.10 Luciferase assay

2T3 cells were selected for this assay based on their low‐endogenous expression of miRNAs. Cells were transfected with 20 ng empty vector, Ccnb1 3′UTR, or MUT Ccnb1 3′UTR for 4 hours in reduced serum and antibiotic‐free Opti‐MEM with Lipofectamine2000. Cells were cotransfected with the pre‐miR‐181c‐5p, inhibitor or a negative control (RiboBio) at a concentration of 20 nmol/L. Cells were harvested for the luciferase assay 48 hours after transfection using a luciferase assay kit (Promega, USA) according to the manufacturer's protocol.^36^, ^39^

### 2.11 Chemicals and reagents

Unless otherwise stated, all chemicals and reagents used in this study were obtained from Sigma Chemical Company.

### 2.12 Statistical analysis

The experimental data were statistically analysed with the SPSS 19.0 software. Data are presented as means ± SD. Unpaired, two‐tailed Student's *t* tests or one‐way analysis of variance was used to compare the means. The test was considered to be significant when *P* < 0.05.

## 3 RESULTS

### 3.1 Simulated microgravity inhibits osteoblasts proliferation

**Figure 1:**
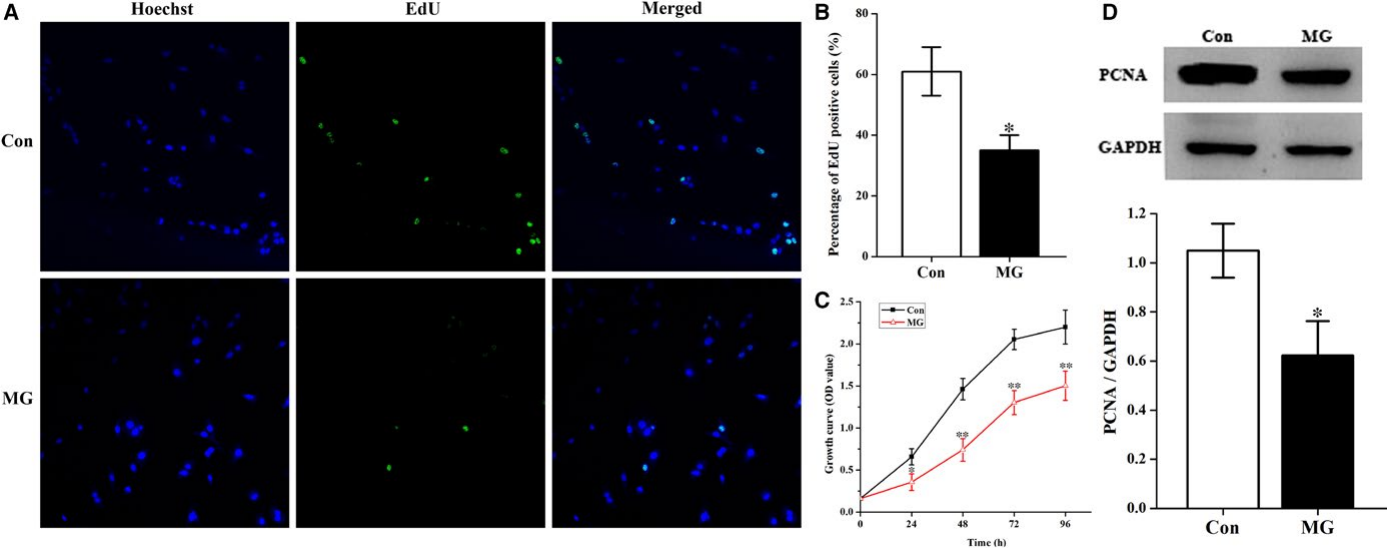
Simulated microgravity (MG) inhibits cell growth of primary mouse osteoblasts. A, EdU incorporation experiments were analysed using an inverted microscope linked to a confocal scanning unit. Proliferating primary mouse osteoblasts were labelled with EdU. Osteoblasts were stained with the nucleic acid dye Hoechst (blue) and EdU (green). B, Histogram of the average percentage of EdU‐positive cells from the two groups. The EdU incorporation rate was expressed as the ratio of EdU‐positive cells to total Hoechst positive cells (n = 4). C, Comparison of changes in cell growth between control (Con) and MG groups. Cells were seeded on 96‐well plates at a density of 2000 cells/well. Cell proliferation was evaluated by a CCK‐8 assay at 24‐96 h (n = 3). D, Western blot analysis of PCNA expression in cells treated with simulated microgravity. The total protein loaded per lane was 40 μg. Detection of GAPDH on the same blots was used to verify equal loading amongst the various lanes (upper). Histogram illustrated average data for the relative expression of PCNA present in cells from each group quantified by camera‐based detection of emitted chemiluminescence (lower) (n = 4). The results were expressed as the mean ± SD. Two‐tailed Student's *t* test was performed for each sample against control samples. *P < 0.05 and **P < 0.01, when compared with the stationary control.

To assess the effects of simulated microgravity on osteoblast proliferation, EdU labelling experiments were performed. As shown in Figure 1A and B, the number of EdU‐positive cells was decreased in cells under the simulated microgravity condition compared with cells in the control group. Subsequently, we employed the cell counting kit‐8 (CCK‐8) assay to further define the role of simulated microgravity in the inhibition of osteoblast proliferation. The time dependent growth curve of cells was shifted downwards after 48 hours treatment with simulated microgravity, indicating that simulated microgravity inhibits osteoblast proliferation (Figure 1C). These results are in agreement with the EdU labelling assay. In addition, PCNA expression analysis was conducted to confirm the influence of simulated microgravity on osteoblast proliferation. Our results showed that there was a decreased expression of PCNA in the simulated microgravity group compared with that of the control group (Figure 1D). These results are in agreement with the previously described experiments.

### 3.2 Simulated microgravity induces osteoblast cell cycle arrest in the G_2_ phase

**Figure 2:**
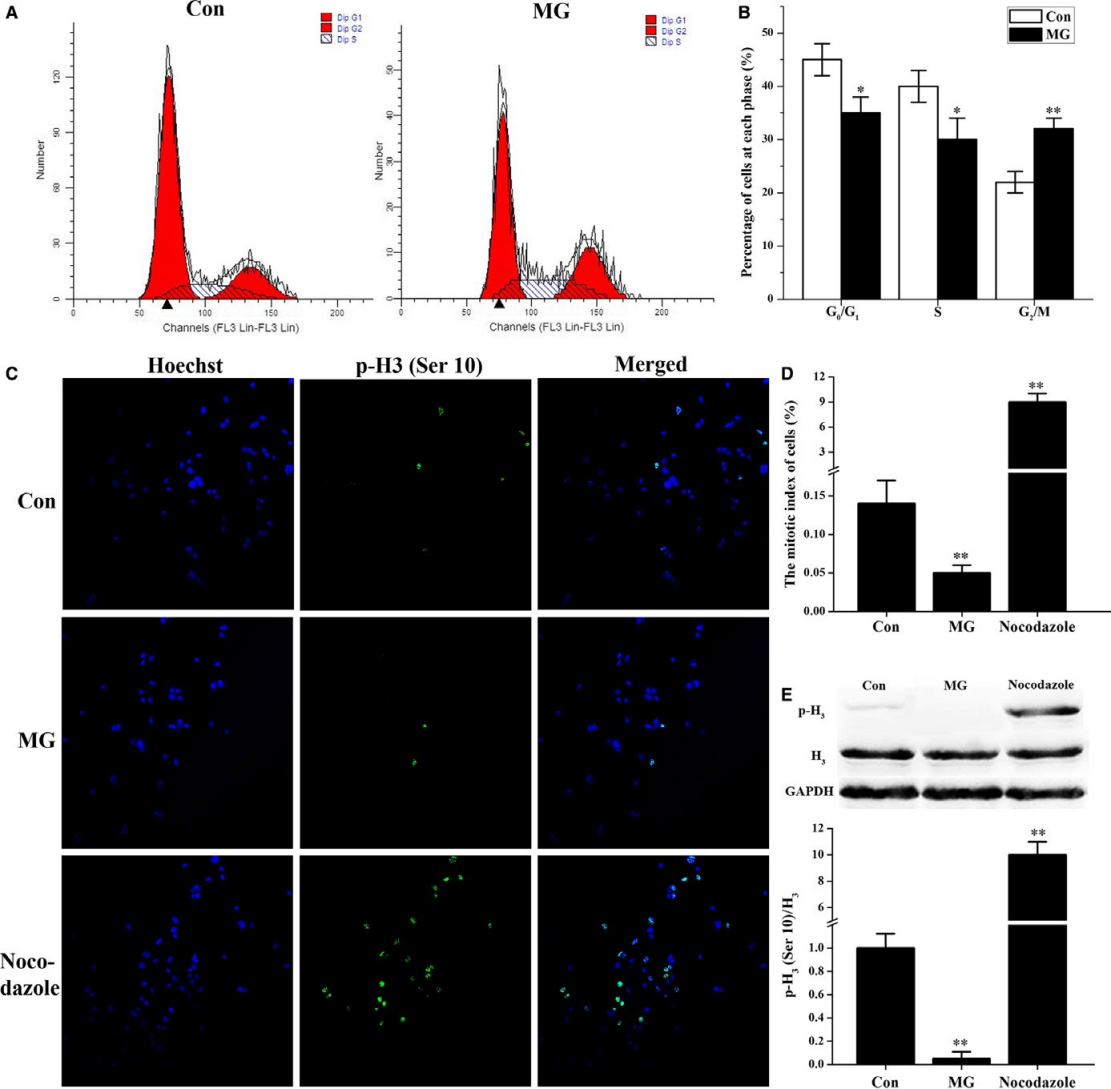
Cell cycle of osteoblasts is arrested in the G_2_ phase (as opposed to the M phase) in response to simulated microgravity. A and B, Flow cytometry analysis of primary mouse osteoblasts treated with simulated microgravity was performed to test the cell cycle distribution. A, Representative histograms indicate the cell cycle distribution in different groups. The relative DNA contents of cells were determined by PI staining. B, The percentage of cells in each cycle stage was quantified (n = 5). C‐E, The effect of simulated microgravity on the mitosis index of osteoblasts was detected by immunofluorescence for histone H_3_ (phospho Ser10). C, Cells were seeded onto glass coverslips and, after simulated microgravity treatment for 48 h, cells were fixed, permeabilized and subjected to staining with Hoechst (blue) to visualize nuclei and with anti‐histone H_3_ (phospho Ser10) primary antibody and Alexa Fluor 488 conjugated secondary antibody (green) to visualize cells undergoing mitosis. Images were analysed using a confocal microscope. D, Histogram of the percentage of histone H_3_ (phospho Ser10)‐positive cells from these groups. The mitotic index was expressed as the ratio of histone H_3_ (phospho Ser10)‐positive cells to total Hoechst positive cells (n = 3). E, Western blot analysis of histone H_3_ (phospho Ser10) expression was determined in cell lysates from primary mouse osteoblasts. The total protein loaded per lane was 40 μg. Detection of GAPDH on the same blots was used to verify equal loading among the various lanes (upper). Histogram of the relative expression of histone H_3_ (phospho Ser10) present in cells from each group quantified by camera‐based detection of emitted chemiluminescence (lower) (n = 4). Cells treated with 0.5 μg/mL nocodazole (a mitotic inhibitor) for 24 h were used as a positive control. The results were expressed as the mean ± SD with a one‐way ANOVA with a SNK‐q test. *P < 0.05 and **P < 0.01, compared with the stationary control.

We performed FCM assays to evaluate the effects of simulated microgravity on cell cycle distribution in primary mouse osteoblasts. The proportion of cells in the G_2_/M phase was increased significantly, while the proportion of cells in the G_0_/G_1_ and S phases was decreased in the simulated microgravity group compared with that in the control group (Figure 2A and B). To further clarify the exact ratio of cells in the M phase, we performed immunofluorescence assays for the expression of histone H_3_ (phospho Ser10). Figure 2C and D illustrated that the mitotic index of osteoblasts was decreased in the simulated microgravity group and was significantly increased in cells pretreated with the mitotic inhibitor nocodazole (which is known to block cell cycle progression in the M phase through disruption of mitotic spindles, and which served as a positive control). Moreover, the expression of histone H_3_ (phospho Ser10) was diminished in the simulated microgravity group and was noticeably increased in the nocodazole group compared with the control group (Figure 2E).

### 3.3 Simulated microgravity has no effects on the cellular localization, expression and activity of Cdc2 kinase

**Figure 3:**
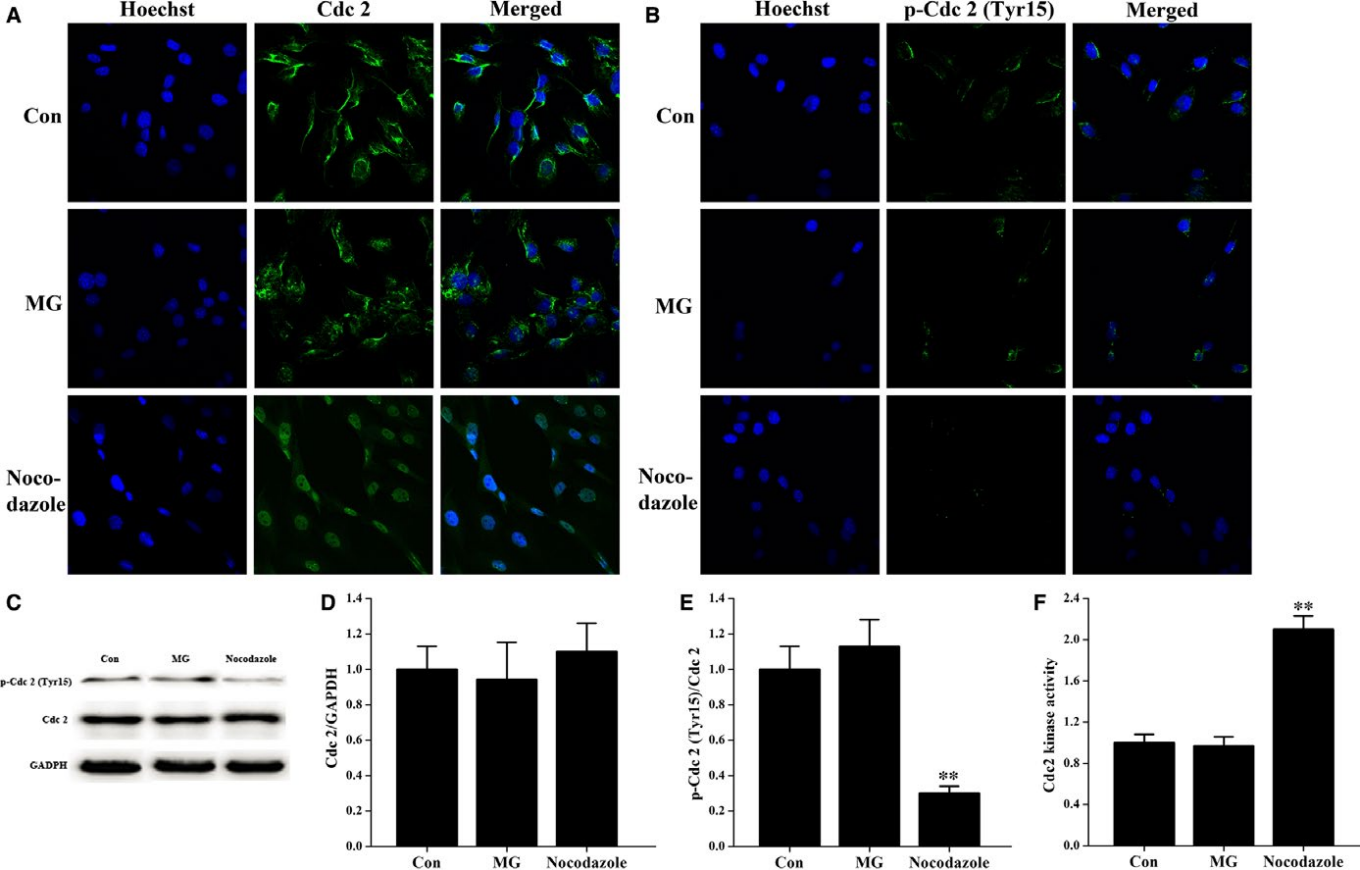
Cellular localization, expression levels and activity of Cdc2 kinase are unchanged under simulated microgravity conditions. A and B, Immunocytochemistry assay was analysed using an inverted microscope linked to a confocal scanning unit (n = 3). Proliferating primary mouse osteoblasts in different groups were stained with the nucleic acid dye Hoechst (blue), anti‐Cdc2 antibody (A) or anti‐p‐Cdc2 antibody (B) and Alexa Fluor 488 conjugated secondary antibody (green). C, Western blot analysis of Cdc2 and p‐Cdc2 expression in cell lysates from osteoblasts in different groups. The total protein loaded per lane was 40 μg. Detection of GAPDH on the same blots was used to verify equal loading among the various lanes. Histogram of the relative expression of Cdc2 (D) and p‐Cdc2 (E) present in cells from the Con, MG and Nocodazole groups quantified by camera‐based detection of emitted chemiluminescence (n = 5). F, Cell lysates obtained from different groups were incubated with the anti‐cyclin B1 antibody reversible immunoprecipitation system. Then, Cdc2‐cyclin B1 kinase activity was measured using a Cdc2‐cyclin B kinase assay kit (n = 4). Nocodazole is a mitotic inhibitor that served as a positive control treatment. Bars represent the mean ± SD with a one‐way ANOVA with a SNK‐q test. **P < 0.01 compared with the stationary control.

In the eukaryotic cell cycle, activation of Cdc2 kinase is required for cells to enter mitosis. We asked whether the simulated microgravity‐induced G_2_ arrest in primary mouse osteoblasts was because of the inactivation of the cyclin B1/Cdc2 kinase complex. As this complex is maintained in an inactive form through phosphorylation of the Cdc2 residues Thr14 and Tyr15, we performed an immunostaining assay to study the cellular localization and expression of Cdc2 and Cdc2 (phospho Tyr15) in osteoblasts under simulated microgravity conditions. As shown in Figure 3A, Cdc2 expression in the control and simulated microgravity groups was localized intracellularly, but was not nuclear. In contrast, Cdc2 had translocated into the nucleus in nocodazole‐stimulated cells (Figure 3A). Interestingly, there was no difference in the cellular localization and fluorescence intensity of Cdc2 in the control and simulated microgravity groups (Figure 3A). Similarly, there was no difference in the cellular localization and expression of Cdc2 (phospho Tyr15) in the control and simulated microgravity groups (Figure 3B), but the expression of Cdc2 (phospho Tyr15) was decreased in cells pretreated with nocodazole (Figure 3B).

To further define the influence of simulated microgravity on expression of Cdc2 kinase, we performed Western blot analysis. Our results demonstrate that the expression of Cdc2 and Cdc2 (phospho Tyr15) in osteoblasts was similar between the control group and the simulated microgravity group (Figure 3C‐E). In addition, we detected a significant decrease in Cdc2 (phospho Tyr15) levels in cells treated with nocodazole (Figure 3C and E). These results are in agreement with the immunostaining staining assay.

We then proceeded to examine Cdc2 activity in osteoblasts under different conditions. Cdc2‐cyclin B1 complexes were isolated from osteoblast lysates by immunoprecipitation with an anti‐cyclin B1 antibody, and Cdc2 kinase activity was determined. We found that there was no difference in Cdc2 kinase activity in the control and simulated microgravity groups (Figure 3F). Additionally, we observed that nocodazole‐treated cells had markedly higher Cdc2 kinase activity than the other two groups (Figure 3F).

### 3.4 Simulated microgravity has no effects on the cellular localization of cyclin B1, but down‐regulates cyclin B1 protein levels

**Figure 4:**
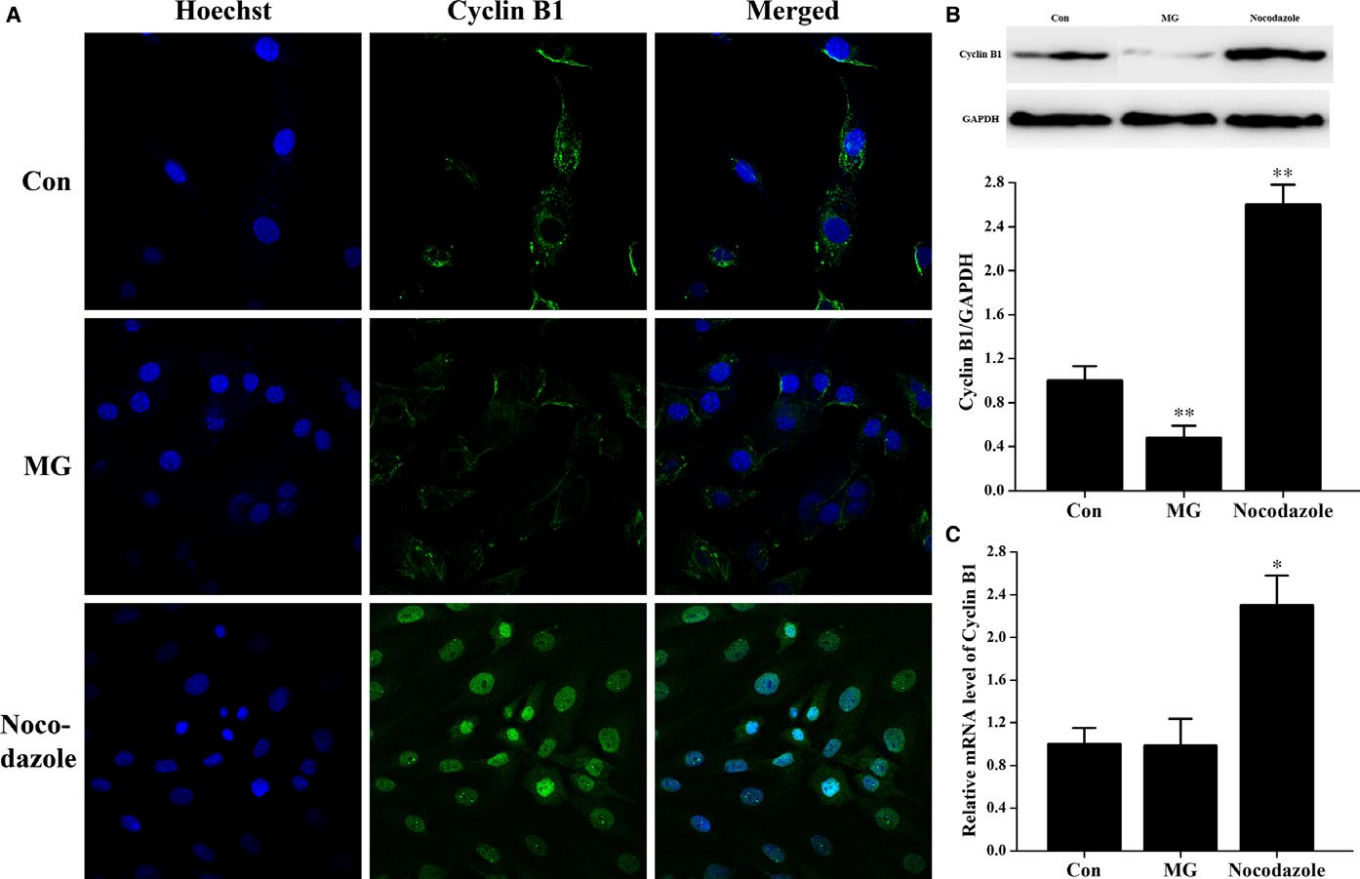
Simulated microgravity does not change the cellular localization of cyclin B1, but decreases its expression. A, Immunostaining staining experiments were conducted using a confocal microscope (n = 3). Primary mouse osteoblasts in different treatment groups were labelled with the nucleic acid dye Hoechst (blue), anti‐cyclin B1 and Alexa Fluor 488 conjugated secondary antibody (green). B, Western blot analysis of cyclin B1 expression in cell lysates from cells in Con, MG and Nocodazole groups. The total protein loaded was 40 μg per lane. Detection of GAPDH on the same blots was used to verify equal loading among the various lanes (upper). Histogram of the relative expression of cyclin B1 present in different conditions quantified by camera‐based detection of emitted chemiluminescence (lower) (n = 3). C, qPCR of relative cyclin B1 mRNA levels in osteoblasts treated with simulated microgravity (n = 6). Primary mouse osteoblasts treated with 0.5 μg/mL nocodazole for 24 h served as a positive control. The results were expressed as the mean ± SD with a one‐way ANOVA with a SNK‐q test. *P < 0.05 and **P < 0.01, compared with the stationary control.

Immunostaining experiments were conducted to test the effects of simulated microgravity on cellular localization of cyclin B1 in osteoblasts. As shown in Figure 4A, cyclin B1 expression in the control and simulated microgravity groups had similar localization patterns, where cyclin B1 was localized intracellularly, but not in the nucleus. In contrast, cyclin B1 translocated into the nucleus in nocodazole‐stimulated cells (Figure 4A). As shown in Figure 4B, the protein levels of cyclin B1 in osteoblasts were decreased in the simulated microgravity group and increased in cells treated with nocodazole. In addition, we performed qPCR to investigate the changes in cyclin B1 transcript levels in the different groups. Cyclin B1 transcript levels in osteoblasts were similar between the control group and the simulated microgravity group (Figure 4C), and nocodazole‐treated cells had higher cyclin B1 mRNA levels than the other two groups (Figure 4C). These data do not agree with the protein data, suggesting that mechanisms at the post‐transcriptional level may play a role in the regulation of cyclin B1 expression.

### 3.5 Overexpression of cyclin B1 rescues the cell cycle arrest in the G_2_ phase and partially recovers the inhibition of osteoblast proliferation induced by simulated microgravity

**Figure 5:**
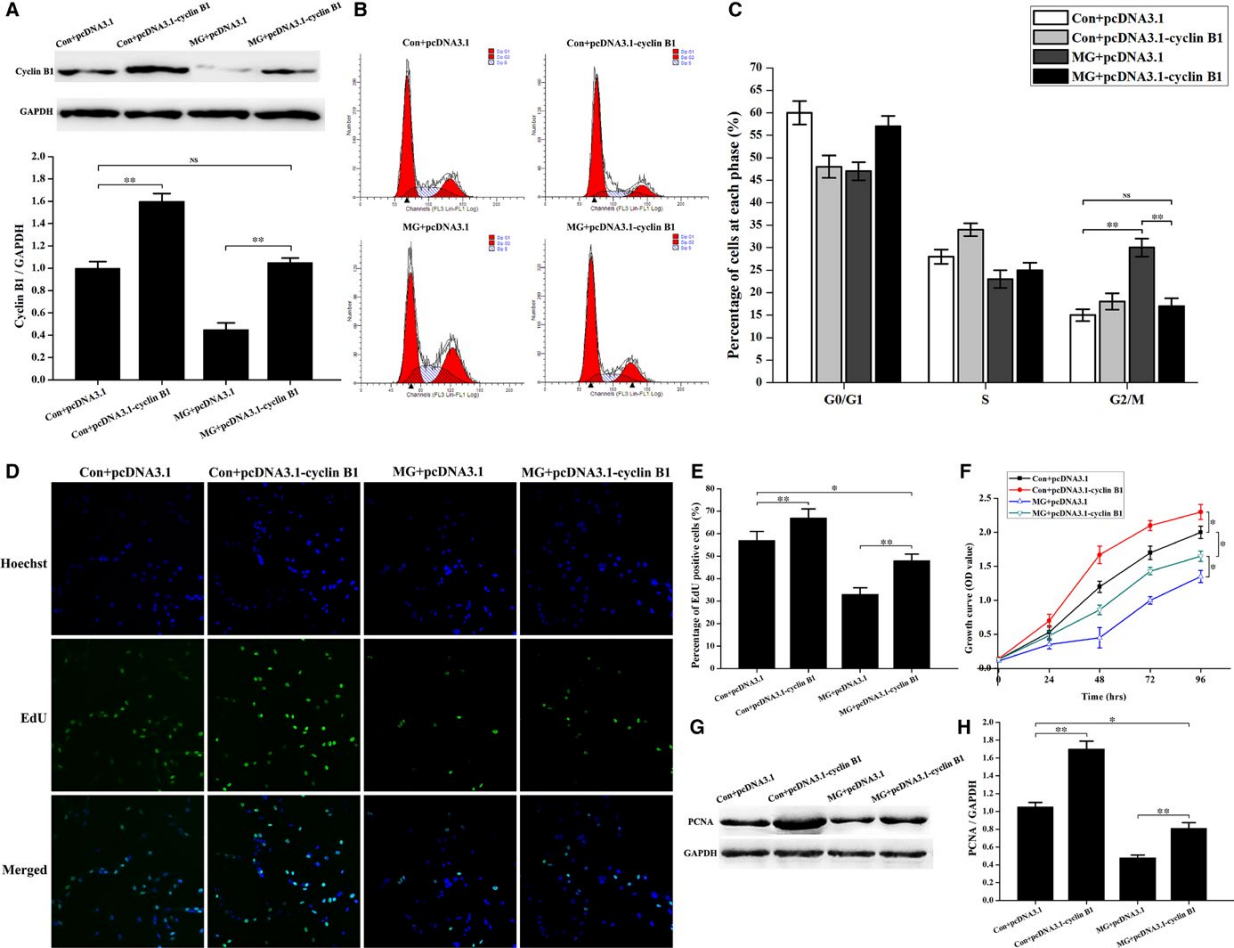
Cyclin B1 overexpression restores the cell cycle arrest in the G_2_ phase and partially counteracts the decrease of osteoblast proliferation induced by simulated microgravity. A, Western blot to test the efficiency of the pcDNA3.1‐cyclin B1 vector in primary mouse osteoblasts under normal gravity and simulated microgravity conditions. Cell lysates were obtained after transfection with pcDNA3.1‐cyclin B1 or pcDNA3.1 empty vector in the Con and MG groups. The total protein loaded was 40 μg per lane. Detection of GAPDH on the same blots was used to verify equal loading among the various lanes (upper). Histogram of the relative expression of cyclin B1 in different treatment groups quantified by camera‐based detection of emitted chemiluminescence (lower) (n = 3). B and C, FCM analyses of osteoblasts transfected with pcDNA3.1‐cyclin B1 or pcDNA3.1 empty vector in the Con and MG groups. B, Representative histograms indicating cell cycle distribution in different groups. The relative DNA content of cells was determined by PI staining. C, The percentage of cells in each cycle stage was quantified (n = 4). D, EdU labelling assays were analysed using an inverted microscope linked to a confocal scanning unit. Proliferating osteoblasts were loaded with EdU. Osteoblasts were stained with the nucleic acid dye Hoechst (blue) and EdU (green). E, Histogram of the percentage of EdU‐positive cells from different groups. The EdU incorporation rate was expressed as the ratio of EdU‐positive cells to total Hoechst positive cells (n = 3). F, Comparison of cell growth changes in different treatment groups. Cells were seeded on 96‐well plates at a density of 2000 cells/well. Cell proliferation was evaluated by a CCK‐8 assay at 24‐96 h (n = 4). G, Western blot analysis of PCNA expression in cells transfected with pcDNA3.1‐cyclin B1 or pcDNA3.1 empty vector in Con and MG groups. The total protein loaded per lane was 40 μg. Detection of GAPDH on the same blots was used to verify equal loading among the various lanes. H, Histogram of the relative expression of PCNA present in cells from each group as quantified by camera‐based detection of emitted chemiluminescence (lower) (n = 4). The results were expressed as the mean ± SD with a one‐way ANOVA with a SNK‐q test. *P < 0.05 and **P < 0.01, compared with the stationary control.

To confirm the effect of cyclin B1 on cell cycle arrest under simulated microgravity conditions, pcDNA3.1‐cyclin B1 or pcDNA3.1 empty vector was transfected into primary mouse osteoblasts, and Western blot analyses and flow cytometer assays were performed. As shown in Figure 5A, pcDNA3.1‐cyclin B1 treatment resulted in increased cyclin B1 in osteoblasts in the altered gravity condition, and there was no difference in cyclin B1 expression between control + pcDNA3.1 and simulated microgravity + pcDNA3.1‐cyclin B1 groups. Then, a flow cytometer assay was conducted to study the effects of cyclin B1 on the cell cycle in cells exposed to different gravity conditions. The proportion of cells in the G_2_/M phase was decreased significantly in the simulated microgravity + pcDNA3.1‐cyclin B1 group compared with that in the simulated microgravity + pcDNA3.1 group, but was similar to the proportion of cells in G_2_/M in the control + pcDNA3.1 group (Figure 5B and C). These data indicated that up‐regulation of cyclin B1 counteracted cell cycle arrest in the G_2_ phase induced by simulated microgravity.

To further study the influence of cyclin B1 on osteoblast proliferation, we performed EdU labeling experiments, CCK‐8 assays and PCNA expression analysis. The number of EdU‐positive cells was increased in cells in the simulated microgravity + pcDNA3.1‐cyclin B1 group compared with that in the simulated microgravity + pcDNA3.1 group, while the number of EdU‐positive cells was decreased compared with the control + pcDNA3.1 group (Figure 5D and E). As shown in Figure 5F, the time dependent growth curve of cells was shifted upwards after transfection with pcDNA3.1‐cyclin B1 under simulated microgravity conditions, but the curve was shifted downwards in the simulated microgravity + pcDNA3.1‐cyclin B1 group compared with that in the control + pcDNA3.1 group (Figure 5F). PCNA expression analysis illustrated that pcDNA3.1‐cyclin B1 up‐regulated the expression of PCNA under simulated microgravity conditions. However, the expression of PCNA was not restored to control + pcDNA3.1 levels. These results were in agreement with our previous experiments, indicating that overexpression of cyclin B1 partially restored osteoblast proliferation under simulated microgravity conditions.

### 3.6 miR‐181c‐5p inhibits cyclin B1 protein expression in osteoblasts under simulated microgravity conditions

**Figure 6:**
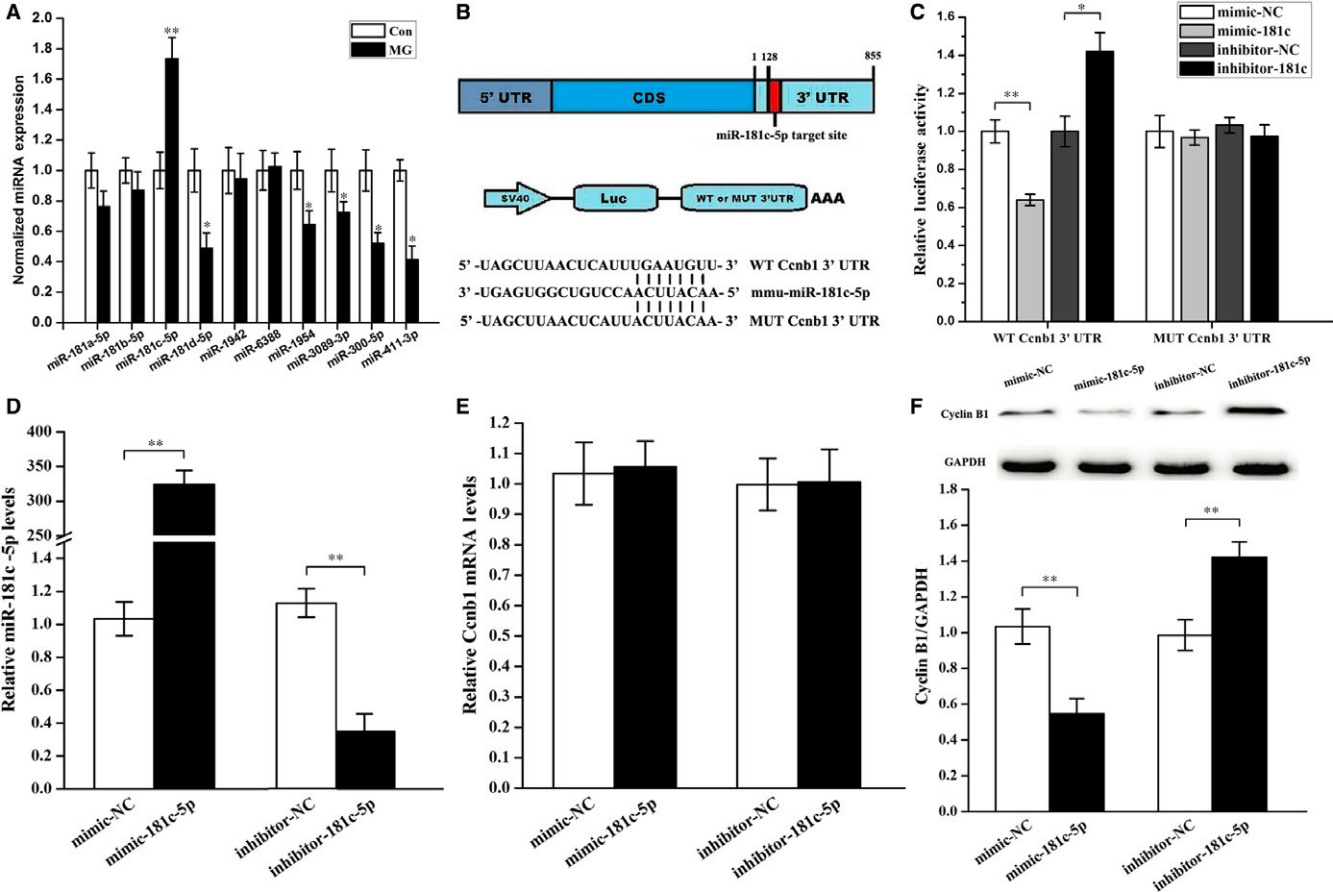
Ccnb1, the gene for cyclin B1, is the target gene of miR‐181c‐5p in primary mouse osteoblasts. miRNA target prediction tools were used to screen for cyclin B1‐targeting miRNAs, and the top ten miRNAs that received the highest composite score were selected for the expression assay. A, qPCR analysis of changes in expression of miR‐181a‐5p, miR‐181b‐5p, miR‐181c‐5p, miR‐181d‐5p, miR‐1942, miR‐6388, miR‐1954, miR‐3089‐3p, miR‐300‐5p and miR‐411‐3p in osteoblasts treated with simulated microgravity (n = 6). B, A schematic illustration of the design of luciferase reporters containing the WT Ccnb1 3′UTR (WT 3′UTR) or the site‐directed mutant Ccnb1 3′UTR (MUT 3′UTR). Sequences below indicate putative miR‐181c‐5p target sites on the WT 3′UTR, the MUT derivative, and the pairing regions of miR‐181c‐5p. C, The effects of the miR‐181c‐5p mimic and inhibitor or their negative controls on the luciferase activity of the WT Ccnb1 3′UTR or MUT Ccnb1 3′UTR reporter in 2 T3 cells. The values in the condition of WT Ccnb1 3′UTR or MUT Ccnb1 3′UTR are shown relative to that of the mimic‐NC in the same condition (n = 3). D, qPCR of miR‐181c‐5p levels in osteoblasts after treatment with mimic‐181c‐5p, inhibitor‐181c‐5p or their negative controls (n = 3). E, qPCR experiments were performed to detect changes in Ccnb1 mRNA expression in osteoblasts after treatment with mimic‐181c‐5p, inhibitor‐181c‐5p or the negative controls (n = 3). F, Western blot analyses of cyclin B1 proteins levels in primary mouse osteoblasts after treatment with mimic‐181c‐5p, inhibitor‐181c‐5p or the negative controls for 48 h. The total protein loaded per lane was 40 μg. Detection of GAPDH on the same blots was used to verify equal loading among the various lanes (upper). The histogram illustrated the relative expression of cyclin B1 present in cells from each group as quantified by camera‐based detection of emitted chemiluminescence (lower) (n = 3). The results were expressed as the mean ± SD with a one‐way ANOVA with a SNK‐q test. *P < 0.05 and **P < 0.01, compared with the stationary control.

To further explore whether miRNAs are important for changes in cyclin B1 expression under simulated microgravity conditions, bioinformatics analysis was performed with TargetScan, miRanda and miRWalk, miRNA target prediction software to screen for cyclin B1‐targeting miRNAs. Based on these analyses, the top ten miRNAs that received the highest composite score were selected for the expression assay. Only miR‐181c‐5p was remarkably up‐regulated in the simulated microgravity group has compared to the control group (Figure 6A). These findings indicate that miR‐181c‐5p may be involved in regulation of cyclin B1 expression under simulated microgravity conditions.

Subsequently, a dual luciferase reporter system was constructed containing either the wild‐type Ccnb1 3′UTR sequence (WT) or an Ccnb1 3′UTR mutant sequence (MUT) to test whether miR‐181c‐5p directly inhibits cyclin B1 protein translation by binding to a predicted target site in the 3′UTR (Figure 6B). The luciferase reporter assay demonstrated that mimic‐181c‐5p decreased WT Ccnb1 3′UTR luciferase reporter activity, whereas inhibitor‐181c‐5p increased WT Ccnb1 3′UTR luciferase reporter activity, but not MUT Ccnb1 3′UTR reporter activity (Figure 6C). By comparison, the miRNA negative control had no effect on luciferase activity when cotransfected with either the Ccnb1 3′UTR or the Ccnb1 3′UTR mutant (Figure 6C). This result suggests that Ccnb1 is a direct target of miR‐181c‐5p.

Moreover, to confirm the effect of miR‐181c‐5p on cyclin B1 expression, miR‐181c‐5p mimic or inhibitor was transfected into primary mouse osteoblasts and qPCR and Western blot analyses were performed to examine the expression of cyclin B1. Intracellular miR‐181c‐5p levels were significantly up‐regulated after mimic treatment and markedly down‐regulated after inhibitor treatment (Figure 6D). As shown in Figure 6E and F, overexpression of miR‐181c‐5p decreased cyclin B1 protein levels, while knockdown of miR‐181c‐5p increased cyclin B1 protein levels, even though the cyclin B1 mRNA levels were only slightly changed.

### 3.7 Inhibition of miR‐181c‐5p partially counteracts cell cycle arrest in the G_2_ phase and inhibition of osteoblast proliferation induced by simulated microgravity

**Figure 7:**
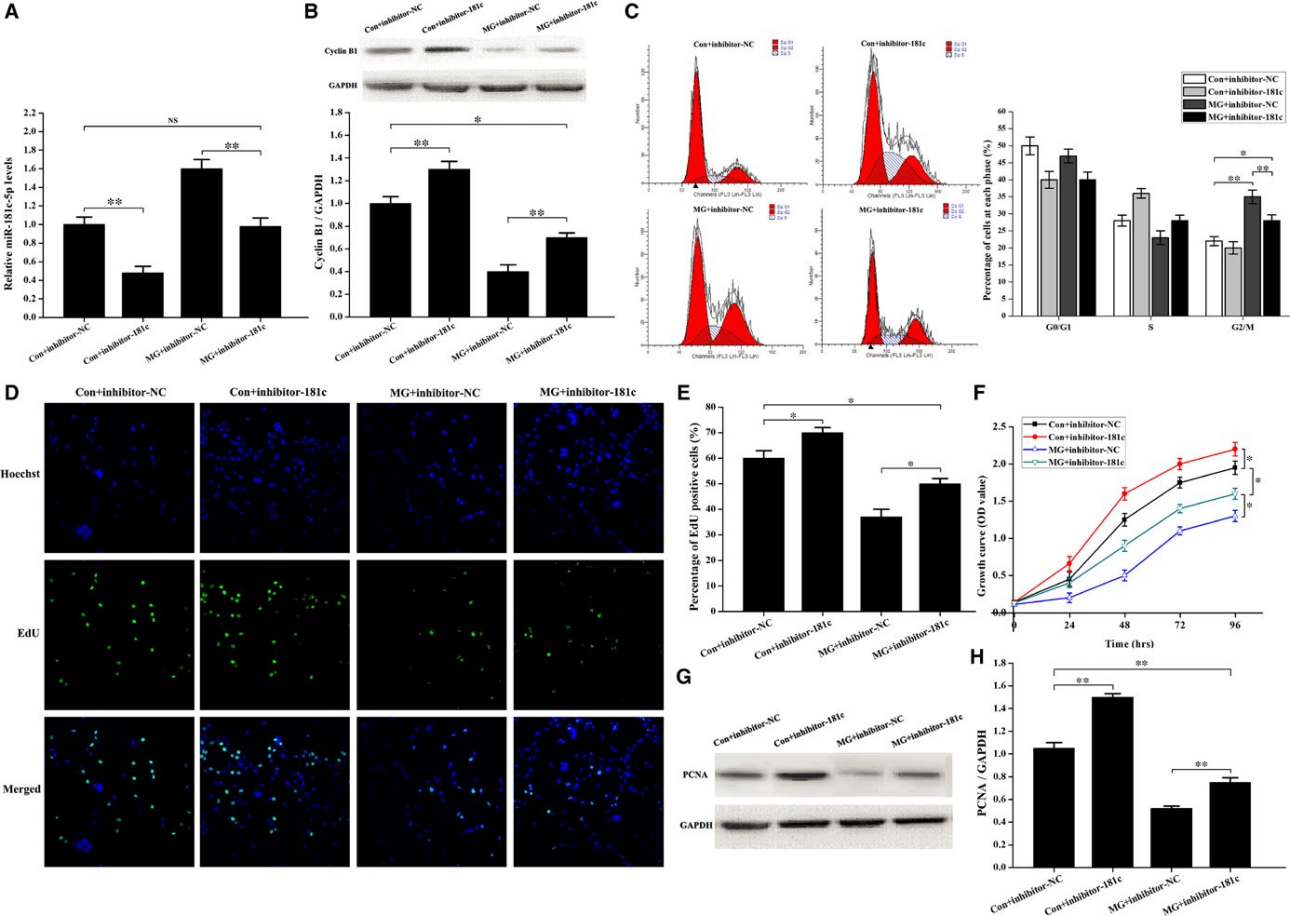
Induction of cell cycle arrest in the G_2_ phase and decreased proliferation in primary mouse osteoblasts treated with simulated microgravity partially depends on the up‐regulation of miR‐181c‐5p. A, qPCR of the miR‐181c‐5p levels in osteoblasts to test the efficiency of inhibitor‐181c‐5p under normal gravity and simulated microgravity conditions (n = 3). B, Western blot experiments in primary mouse osteoblasts test the effects of inhibitor‐181c‐5p on cyclin B1 expression in the Con and MG groups. Cell lysates were obtained after transfection with inhibitor‐181c‐5p or inhibitor NC in both groups. The total protein loaded was 40 μg per lane. Detection of GAPDH on the same blots was used to verify equal loading among the various lanes (upper). The histogram showed the relative expression of cyclin B1 present in different groups as quantified by camera‐based detection of emitted chemiluminescence (lower) (n = 3). C, FCM analyses of osteoblasts transfected with inhibitor‐181c‐5p or inhibitor NC in Con and MG groups to examine the cell cycle distribution. Representative histograms indicate the cell cycle distribution in the different groups. The relative DNA content of cells was determined by PI staining (left). The percent of cells in each cycle stage was quantified and showed as histograms (right) (n = 4). D, EdU labelling assays were analysed using an inverted microscope linked to a confocal scanning unit. Proliferating osteoblasts were loaded with EdU. Osteoblasts were stained with the nucleic acid dye Hoechst (blue) and EdU (green). E, Histogram of the percentage of EdU‐positive cells from different groups. The EdU incorporation rate was expressed as the ratio of EdU‐positive cells to total Hoechst positive cells (n = 3). F, Comparison of changes in cell growth among the different groups. Cells were seeded on 96‐well plates at a density of 2000 cells/well. Cell proliferation was evaluated by a CCK‐8 assay at 24‐96 h (n = 3). G, Western blot of PCNA expression in cells transfected with inhibitor‐181c‐5p or inhibitor NC in the Con and MG groups. The total protein loaded per lane was 40 μg. Detection of GAPDH on the same blots was used to verify equal loading among the various lanes. H, Histogram of the relative expression of PCNA present in cells from each group as quantified by camera‐based detection of emitted chemiluminescence (lower) (n = 4). The results were expressed as the mean ± SD with a one‐way ANOVA with a SNK‐q test. *P < 0.05 and **P < 0.01, compared with the stationary control.

To assess the effect of miR‐181c‐5p on cyclin B1 expression and the cell cycle under simulated microgravity conditions, miR‐181c‐5p inhibitor or negative control inhibitor was transfected into primary mouse osteoblasts, and qPCR, Western blot analyses and flow cytometer assays were performed. We initially examined the efficiency of the miR‐181c‐5p inhibitor under normal gravity and simulated microgravity conditions. In cells transfected with miR‐181c‐5p inhibitor, the expression of miR‐181c‐5p was significantly down‐regulated in the different gravity condition, but there was no difference between the control + inhibitor‐NC and simulated microgravity + inhibitor‐181c groups (Figure 7A). Then, Western blot analyses were conducted to study the effects of miR‐181c‐5p inhibitor on cyclin B1 expression in different gravity conditions. As shown in Figure 7B, the expression of cyclin B1 was increased after transfection with miR‐181c‐5p inhibitor under normal gravity and simulated microgravity conditions (Figure 7B). However, the expression of cyclin B1 was decreased in the simulated microgravity + inhibitor‐181c group compared with the control + inhibitor‐NC group (Figure 7B). Moreover, a similar trend was observed for the effects of miR‐181c‐5p in regulation of cell cycle arrest induced by simulated microgravity. Flow cytometer assay demonstrated that the proportion of cells in the G_2_/M phase was decreased significantly in the simulated microgravity + inhibitor‐181c group compared with that in the simulated microgravity + inhibitor‐NC group, but was increased compared to that in the control + inhibitor‐NC group (Figure 7C). Our data indicate that inhibition of miR‐181c‐5p partially counteracts cell cycle arrest in the G_2_ phase induced by simulated microgravity.

To further study the influence of miR‐181c‐5p on osteoblast proliferation, we performed EdU labelling experiments, CCK‐8 assays and PCNA expression analysis. As shown in Figure 7D and E, the number of EdU‐positive cells was increased in the simulated microgravity + inhibitor‐181c group compared with the simulated microgravity + inhibitor‐NC group, but the number of EdU‐positive cells was decreased compared with the control + inhibitor‐NC group. Furthermore, the time dependent growth curve of cells was shifted upwards after transfection with miR‐181c‐5p inhibitor under simulated microgravity conditions. However, the curve was shifted downwards in the simulated microgravity + inhibitor‐181c group compared with the control + inhibitor‐NC group (Figure 7F). Similar results were observed for PCNA protein levels, where Western blot identified that the transfection of miR‐181c‐5p inhibitor rescued the expression of PCNA under simulated microgravity conditions, but these levels were not restored to control + inhibitor‐NC levels (Figure 7G and H). We then transfected miR‐181c‐5p mimic and further evaluated the effect of miR‐181c‐5p on cell cycle and osteoblast proliferation (Figure S2). These results are in agreement with our previous experiments, and taken together, our data indicate that down‐regulation of miR‐181c‐5p partially counteracts the inhibition of osteoblast proliferation under simulated microgravity conditions.

## 4 DISCUSSION

In this study, we found that simulated microgravity inhibits cell proliferation and induces cell cycle to be arrested in the G_2_ phase in primary mouse osteoblasts. Furthermore, we demonstrated that cell cycle arrest in osteoblasts caused by simulated microgravity is mainly because of the decreased cyclin B1 expression. Lastly, we showed that miR‐181c‐5p directly inhibits cyclin B1 protein translation by binding to a target site in the 3′UTR, and this is partially responsible for the cell cycle arrest and cell proliferation inhibition in osteoblasts under simulated microgravity conditions. Our study demonstrates that simulated microgravity inhibits cell proliferation and induces cell cycle arrest in the G_2_ phase in primary mouse osteoblasts partially through the miR‐181c‐5p/cyclin B1 pathway.

Orbital spaceflight has clearly demonstrated that the absence or the reduction of gravity has significantly adverse effects on astronauts.^8^, ^44^ The skeletal deconditioning, such as reduced bone mass, altered mineralization patterns and decreased expression of bone matrix genes, has been described in astronauts or animal models under microgravity conditions.^44^, ^45^, ^46^, ^47^ Impairment of the skeletal system induced by mechanical unloading has been known as one of the main limitations of long‐term spaceflight, and this problem has received general concern by researchers.^8^, ^44^ Unfortunately, as a result of the expensive and limited nature of spaceflight missions, conducting in vitro studies under true microgravity conditions are both impractical and difficult. Thus, several ground‐based rotational devices (in particular, clinostats) have been developed to simulate microgravity for cell‐based studies. These devices simulate microgravity by constantly rotating around at least one axis to produce a vector‐averaged gravity such that cells have insufficient time to sense the gravity vector. Exposure to such rotational systems significantly inhibits the proliferation, differentiation and mineralization of osteoblasts, thereby exerting similar effects to true microgravity on osteoblasts.^38^, ^40^

Decreased osteoblast proliferation is thought to play a central role in microgravity‐induced bone loss.^40^, ^45^, ^48^ Our previous study has revealed that simulated microgravity inhibits cell proliferation in MC3T3‐E1 pre‐osteoblasts.^40^ Several studies have reported that skeletal unloading leads to bone loss, at least in part because of the disrupted insulin‐like growth factor, and results in reduced osteoblast proliferation and differentiation.^49^ In the present study, we confirmed that impaired cell proliferation is observed in primary mouse osteoblasts under simulated microgravity conditions.

It is generally accepted that induction of cell cycle arrest is a main reason for abnormal cell proliferation.^9^, ^10^, ^11^ Therefore, we evaluated the effects of simulated microgravity on the cell cycle distribution of primary mouse osteoblasts in vitro in this study. Flow cytometry results revealed that simulated microgravity significantly increased the proportion of cells in the G_2_/M phase. Further analysis confirmed that cells in the M phase were decreased significantly under simulated microgravity conditions using immunofluorescence staining and Western blot analysis of phospho‐histone H_3_. Thus, it is clear that simulated microgravity could induce primary mouse osteoblast cell cycle arrest in the G_2_ phase. These observations are consistent with previous studies. Cogoli‐Greuter et al reported that mechanical unloading inhibits cell cycle progression by arresting cells in the G_2_/M phase in leucocytes.^14^ Yan et al illustrated that clinorotation could inhibit proliferation in bone marrow mesenchymal stem cells because of the arrested cell cycle in G_2_/M.^15^ Benavides et al demonstrated that simulated microgravity slowed the proliferation of myoblasts by arresting their exit from the G_2_/M phase of the cell cycle.^12^ There are also reports, however, which demonstrate that microgravity induces cell cycle arrest in other phases. For example, Kamal et al demonstrated that the number of cells in the S phase increased significantly under simulated microgravity conditions in Arabidopsis cells, which led to a reduction of the proportion of cells in G_1_.^13^ Dai et al showed that simulated microgravity inhibits population growth of bone marrow mesenchymal stem cells and causes cell cycle arrest in the G_0_/G_1_ phase.^16^ This variation in results may be because of the different species and sources of cells, or the different ground‐based rotational devices.

In eukaryote cells, the cyclin B1‐Cdc2 kinase complex plays a central role in cell cycle transition from the G_2_ phase into mitosis.^19^ Tran et al reported that fibroblast growth factors inhibit the activity of the Cdc2 kinase complex to induce a transient G_2_ arrest in chondrocytes.^50^ Others have shown that sterigmatocystin induces cell cycle arrest in the G_2_phase in human gastric epithelium cells partially through the down‐regulation of Cdc2 kinase expression and activity.^18^ Li et al demonstrated that Cdc2 kinase complex activity is decreased in human herpesvirus 6‐infected T cells leading to cell cycle arrest in the G_2_ phase.^17^ Intriguingly, in this study, we found that simulated microgravity induces primary mouse osteoblast cell cycle arrest in the G_2_ phase, but has no effects on the cellular localization, expression and activity of Cdc2 kinase. The activity of Cdc2 kinase is also regulated by the activation of its cyclin subunits. Thus, we further tested the effects of simulated microgravity on cellular localization and the expression of cyclin B1 in osteoblasts. Our findings showed that simulated microgravity has no effects on the cellular localization of cyclin B1, but markedly suppresses its expression in primary mouse osteoblasts. Then, we examined cell cycle distribution and cell proliferation following the up‐regulation of cyclin B1 expression to confirm that the reduction of cyclin B1 was involved in the cell cycle arrest and decreased osteoblast proliferation in primary mouse osteoblasts under simulated microgravity. Our results demonstrated that the overexpression of cyclin B1 rescues the cell cycle arrest in the G_2_ phase and partially recovers the inhibition of osteoblast proliferation induced by simulated microgravity. This is consistent with other observations that overexpression of cyclin B1 in human oesophageal squamous cell carcinoma cells enhances cell proliferation and invasion in vitro and in vivo.^51^, ^52^ In this study, we found that overexpression of cyclin B1 showed no effects on G_2_ phase in the normal condition; it decreased the proportion of cells in the G_1_ phase and increased the proportion of cells in the S phase. One explanation could be that there are appropriate and proper proportions of cyclin B1 and Cdc2. Even if we up‐regulated the cyclin B1 expression, there may be not enough active Cdc2 to combine with and to form the cyclin B1‐Cdc2 kinase complex in the normal condition. Another possible explanation could be that the mechanisms of cyclin B1 in regulating cell cycle are very complicated in primary mouse osteoblasts. Our data suggested that the cell cycle arrest and impaired osteoblast proliferation in primary mouse osteoblasts under simulated microgravity conditions could be attributed to decreased cyclin B1 protein levels.

There are many factors can regulate the expression of cyclin B1. Recent works have shown that minute virus of mice infection reduced the protein and RNA levels of cyclin B1 by targeting FoxM1 in human NB324K and murine A9 cells.^26^ Others have reported that long non‐coding RNA 00312 down‐regulates cyclin B1 and inhibits hepatocellular carcinoma cell proliferation in vitro and in vivo.^25^ Zhang et al demonstrated that IL‐18 can augment cell proliferation via the p38/ATF2 pathway by targeting cyclin B1, cyclin B2, cyclin A2 and Bcl‐2 in BRL‐3A rat liver cells.^24^ These experiments implied that changes in cyclin B1 expression induced by different factors coincide with the alteration of cyclin B1 mRNA. However, our findings show unchanged expression of cyclin B1 mRNA levels, which does not correlate with the decreased expression of cyclin B1 protein levels in primary mouse osteoblasts under simulated microgravity. Therefore, this result indicated that a post‐transcriptional regulation mechanism may take part in regulating the expression of cyclin B1 protein.

The small non‐coding RNA molecules miRNAs function in RNA silencing and post‐transcriptional regulation of gene expression. Recently, three miRNAs have been linked to regulation of cyclin B1 expression under different experimental conditions using luciferase‐based reporter assays. Kim et al have shown that Ccnb1 is a target of miR‐199a‐5p in mouse keratinocytes.^33^ Others have validated that Ccnb1 is target of miRNA‐410 as its overexpression reduces Ccnb1 protein levels and decreases cell proliferation of gonadotroph tumours.^34^ Khan et al reported that miR‐379 regulates cyclin B1 expression and is decreased in breast cancer.^35^ To further explore whether a miRNA family is important for the alteration of cyclin B1 expression in primary mouse osteoblasts under simulated microgravity conditions, a bioinformatics analysis was performed with TargetScan, miRanda and miRWalk, the miRNA target prediction software, to screen for cyclin B1‐targeting miRNAs. Based on these analyses, the top ten miRNAs that received the highest composite score were selected for the expression assay. We examined all ten miRNAs by qPCR to screen which miRNAs may affect cyclin B1 expression in osteoblasts under simulated microgravity conditions. Our results showed that simulated microgravity only increases the expression of miR‐181c‐5p, indicating that miR‐181c‐5p may be involved in regulation of cyclin B1 expression under simulated microgravity conditions.

Some aspects of the mechanisms by which miR‐181c regulates gene expression are already known. Specifically, miR‐181c promotes high‐glucose‐induced dysfunction in human umbilical vein endothelial cells by targeting leukaemia inhibitory factor.^53^ Furthermore, miR‐181c protects CsA‐induced renal damage and fibrosis through inhibition of epithelial‐mesenchymal transition.^54^ In addition, in non‐small cell lung cancer cells, miR‐181c contributes to cisplatin resistance by targeting Wnt inhibition factor 1.^55^ In this study, we confirmed that Ccnb1 is a new target gene of miR‐181c‐5p in primary mouse osteoblasts, as indicated by a luciferase assay. Furthermore, miR‐181c‐5p negatively regulated cyclin B1 expression at the post‐transcriptional level. These data identify a novel mechanism and a target site for miR‐181c‐5p that warrants further study. To further uncover the role of miR‐181c‐5p in regulating cyclin B1 expression, cell cycle progression and cell proliferation under simulated microgravity conditions, we tested the effect of miR‐181c‐5p inhibitor in primary mouse osteoblasts. Our data suggested that the up‐regulation of miR‐181c‐5p in simulated microgravity is, at least in part, involved in the regulation of cyclin B1 expression, cell cycle arrest and impaired cell proliferation in osteoblasts under simulated microgravity conditions.

It should be noted that there are some limitations to our study. We noticed that miR‐181c‐5p inhibitor partially counteracts the decreased cyclin B1 expression, cell cycle arrest and inhibition of osteoblast proliferation induced by simulated microgravity. However, other underlying mechanisms for regulation of cyclin B1 expression, cell cycle distribution and cell proliferation in osteoblasts that experience mechanical unloading remain to be investigated. Additionally, we have tested the role of miR‐181c‐5p in osteoblast proliferation, but whether our findings can be extended to osteogenic differentiation and mineralization warrants further studies. Furthermore, the in vitro results obtained using primary mouse osteoblasts in this study have not yet been confirmed in vivo. Accordingly, we plan to confirm our findings in vivo in a future study.

In summary, our study provides a new finding that simulated microgravity inhibits cell proliferation and induces cell cycle arrest in the G_2_ phase in primary mouse osteoblasts via suppression of cyclin B1 expression. Moreover, down‐regulation of cyclin B1 expression, cell cycle arrest and inhibition of osteoblast proliferation is partially related to the up‐regulation of miR‐181c‐5p, which is induced by simulated microgravity. This work may provide a novel mechanism of microgravity‐induced adverse effects on osteoblasts, which offers a new avenue to further investigate bone loss induced by microgravity.

## CONFLICTS OF INTEREST

The authors declare that they have no conflicts of interest with the contents of this article.

## AUTHOR CONTRIBUTION

Study design: ZS, SZ, FS and JZ; Data collection: YL, HW, MC, SG, JL and LT; Contribution of new reagents or analytical tools: YL, HW, ZH, YW and KW; Data analysis: LZ, XC, and ZS; Manuscript preparation: ZS, YL, HW, SZ, FS and JZ.

## References

[1] Aisha MD , Nor‐Ashikin MN , Sharaniza AB , et al. Orbital fluid shear stress promotes osteoblast metabolism, proliferation and alkaline phosphates activity in vitro. Exp Cell Res. 2015;337:87‐93. 26163894 10.1016/j.yexcr.2015.07.002

[2] Saito M , Soshi S , Fujii K . Effect of hyper‐ and microgravity on collagen post‐translational controls of MC3T3‐E1 osteoblasts. J Bone Miner Res. 2003;18:1695‐1705. 12968680 10.1359/jbmr.2003.18.9.1695

[3] Rittweger J , Frost HM , Schiessl H , et al. Muscle atrophy and bone loss after 90 days' bed rest and the effects of flywheel resistive exercise and pamidronate: results from the LTBR study. Bone. 2005;36:1019‐1029. 15811637 10.1016/j.bone.2004.11.014

[4] Bucaro MA , Fertala J , Adams CS , et al. Bone cell survival in microgravity: evidence that modeled microgravity increases osteoblast sensitivity to apoptogens. Ann NY Acad Sci. 2004;1027:64‐73. 15644346 10.1196/annals.1324.007

[5] Duncan RL , Turner CH . Mechanotransduction and the functional response of bone to mechanical strain. Calcif Tissue Int. 1995;57:344‐358. 8564797 10.1007/BF00302070

[6] Fushiki R , Mayahara K , Ogawa M , et al. High‐magnitude mechanical strain inhibits the differentiation of bone‐forming rat calvarial progenitor cells. Connect Tissue Res. 2015;56:336‐341. 25943460 10.3109/03008207.2015.1040878

[7] Wronski TJ , Morey ER . Effect of spaceflight on periosteal bone formation in rats. Am J Physiol. 1983;244:305‐309. 10.1152/ajpregu.1983.244.3.R305 6402940

[8] Morey ER , Baylink DJ . Inhibition of bone formation during space flight. Science. 1978;201:1138‐1141. 150643 10.1126/science.150643

[9] Gutgemann I , Lehman NL , Jackson PK , Longacre TA . Emi1 protein accumulation implicates misregulation of the anaphase promoting complex/cyclosome pathway in ovarian clear cell carcinoma. Mod Pathol. 2008;21:445‐454. 18204430 10.1038/modpathol.3801022

[10] Jayshree RS , Sreenivas A , Tessy M , Krishna S . Cell intrinsic & extrinsic factors in cervical carcinogenesis. Indian J Med Res. 2009;130:286‐295. 19901438

[11] Messner DJ , Kowdley KV . Neoplastic transformation of rat liver epithelial cells is enhanced by non‐transferrin‐bound iron. BMC Gastroenterol. 2008;8:2. 18254965 10.1186/1471-230X-8-2 PMC2275280

[12] Benavides DT , Franco‐Obregon A , Egli M . Gravitational force modulates G2/M phase exit in mechanically unloaded myoblasts. Cell Cycle. 2013;12:3001‐3012. 23974110 10.4161/cc.26029 PMC3875675

[13] Kamal KY , Herranz R , van Loon J , Medina FJ . Simulated microgravity, Mars gravity, and 2 g hypergravity affect cell cycle regulation, ribosome biogenesis, and epigenetics in Arabidopsis cell cultures. Sci Rep. 2018;8:6424. 29686401 10.1038/s41598-018-24942-7 PMC5913308

[14] Cogoli‐Greuter M , Meloni MA , Sciola L , et al. Movements and interactions of leukocytes in microgravity. J Biotechnol. 1996;47:279‐287. 8987569 10.1016/0168-1656(96)01380-6

[15] Yan M , Wang Y , Yang M , et al. The effects and mechanisms of clinorotation on proliferation and differentiation in bone marrow mesenchymal stem cells. Biochem Biophys Res Commun. 2015;460:327‐332. 25804637 10.1016/j.bbrc.2015.03.034

[16] Dai ZQ , Wang R , Ling SK , et al. Simulated microgravity inhibits the proliferation and osteogenesis of rat bone marrow mesenchymal stem cells. Cell Prolif. 2007;40:671‐684. 17877609 10.1111/j.1365-2184.2007.00461.x PMC6496371

[17] Li L , Gu B , Zhou F , et al. Human herpesvirus 6 suppresses T cell proliferation through induction of cell cycle arrest in infected cells in the G2/M phase. J Virol. 2011;85:6774‐6783. 21525341 10.1128/JVI.02577-10 PMC3126536

[18] Xing X , Wang J , Xing LX , et al. Involvement of MAPK and PI3K signaling pathway in sterigmatocystin‐induced G2 phase arrest in human gastric epithelium cells. Mol Nutr Food Res. 2011;55:749‐760. 21287681 10.1002/mnfr.201000344

[19] Castedo M , Perfettini JL , Roumier T , Kroemer G . Cyclin‐dependent kinase‐1: linking apoptosis to cell cycle and mitotic catastrophe. Cell Death Differ. 2002;9:1287‐1293. 12478465 10.1038/sj.cdd.4401130

[20] Russell P , Nurse P . Negative regulation of mitosis by wee1+, a gene encoding a protein kinase homolog. Cell. 1987;49:559‐567. 3032459 10.1016/0092-8674(87)90458-2

[21] Mueller PR , Coleman TR , Kumagai A , Dunphy WG . Myt1: a membrane‐associated inhibitory kinase that phosphorylates Cdc2 on both threonine‐14 and tyrosine‐15. Science. 1995;270:86‐90. 7569953 10.1126/science.270.5233.86

[22] Strausfeld U , Labbe JC , Fesquet D , et al. Dephosphorylation and activation of a p34cdc2/cyclin B complex in vitro by human CDC25 protein. Nature. 1991;351:242‐245. 1828290 10.1038/351242a0

[23] Nurse P . Universal control mechanism regulating onset of M‐phase. Nature. 1990;344:503‐508. 2138713 10.1038/344503a0

[24] Zhang J , Pan C , Xu T , et al. Interleukin 18 augments growth ability via NF‐kappaB and p38/ATF2 pathways by targeting cyclin B1, cyclin B2, cyclin A2, and Bcl‐2 in BRL‐3A rat liver cells. Gene. 2015;563:45‐51. 25752290 10.1016/j.gene.2015.03.010

[25] Wu J , Zhou X , Fan Y , et al. Long non‐coding RNA 00312 downregulates cyclin B1 and inhibits hepatocellular carcinoma cell proliferation in vitro and in vivo. Biochem Biophys Res Commun. 2018;497:173‐180. 29432732 10.1016/j.bbrc.2018.02.049

[26] Fuller MS , Majumder K , Pintel DJ . Minute virus of mice inhibits transcription of the cyclin B1 gene during infection. J Virol. 2017;91:e00428‐e517. 28446681 10.1128/JVI.00428-17 PMC5487563

[27] Ambros V . The functions of animal microRNAs. Nature. 2004;431:350‐355. 15372042 10.1038/nature02871

[28] Bartel DP . MicroRNAs: genomics, biogenesis, mechanism, and function. Cell. 2004;116:281‐297. 14744438 10.1016/s0092-8674(04)00045-5

[29] Kosik KS . MicroRNAs and cellular phenotypy. Cell. 2004;143:21‐26. 10.1016/j.cell.2010.09.008 20887887

[30] Bartel DP . MicroRNAs: target recognition and regulatory functions. Cell. 2009;136:215‐233. 19167326 10.1016/j.cell.2009.01.002 PMC3794896

[31] He L , Hannon GJ . MicroRNAs: small RNAs with a big role in gene regulation. Nat Rev Genet. 2004;5:522‐531. 15211354 10.1038/nrg1379

[32] Zamore PD , Haley B . Ribo‐gnome: the big world of small RNAs. Science. 2005;309:1519‐1524. 16141061 10.1126/science.1111444

[33] Kim BK , Kim I , Lee AR , et al. Mouse‐specific up‐regulation of Ccnb1 expression by miR‐199a‐5p in keratinocyte. Febs Open Bio. 2016;6:1131‐1140. 10.1002/2211-5463.12133 PMC5095150 27833853

[34] Mussnich P , Raverot G , Jaffrain‐Rea ML , et al. Downregulation of miR‐410 targeting the cyclin B1 gene plays a role in pituitary gonadotroph tumors. Cell Cycle. 2015;14:2590‐2597. 26125663 10.1080/15384101.2015.1064207 PMC4614981

[35] Khan S , Brougham CL , Ryan J , et al. miR‐379 regulates cyclin B1 expression and is decreased in breast cancer. PLoS ONE. 2013;8:e68753. 23874748 10.1371/journal.pone.0068753 PMC3707961

[36] Hu Z , Wang Y , Sun Z , et al. miRNA‐132‐3p inhibits osteoblast differentiation by targeting Ep300 in simulated microgravity. Sci Rep. 2015;5:18655. 26686902 10.1038/srep18655 PMC4685444

[37] Sun Z , Li Y , Zhou H , et al. Simulated microgravity reduces intracellular‐free calcium concentration by inhibiting calcium channels in primary mouse osteoblasts. J Cell Biochem. 2019;120:4009‐4020. 30260002 10.1002/jcb.27685

[38] Sun Z , Cao X , Zhang Z , et al. Simulated microgravity inhibits L‐type calcium channel currents partially by the up‐regulation of miR‐103 in MC3T3‐E1 osteoblasts. Sci Rep. 2015;5:8077. 25627864 10.1038/srep08077 PMC4308706

[39] Wang H , Sun Z , Wang Y , et al. miR‐33‐5p, a novel mechano‐sensitive microRNA promotes osteoblast differentiation by targeting Hmga2. Sci Rep. 2016;6:23170. 26980276 10.1038/srep23170 PMC4793269

[40] Sun Z , Cao X , Hu Z , et al. MiR‐103 inhibits osteoblast proliferation mainly through suppressing Cav1.2 expression in simulated microgravity. Bone. 2015;76:121‐128. 25868801 10.1016/j.bone.2015.04.006

[41] Guo T , Wang W , Zhang H , et al. ISL1 promotes pancreatic islet cell proliferation. PLoS ONE. 2011;6:e22387. 21829621 10.1371/journal.pone.0022387 PMC3150357

[42] O'Neill CA , Galasko CS . Calcium mobilization is required for spreading in human osteoblasts. Calcif Tissue Int. 2000;67:53‐59. 10908414 10.1007/s00223001097

[43] Morita E , Tada K , Chisaka H , et al. Human parvovirus B19 induces cell cycle arrest at G (2) phase with accumulation of mitotic cyclins. J Virol. 2001;75:7555‐7563. 11462027 10.1128/JVI.75.16.7555-7563.2001 PMC114990

[44] Vico L , Collet P , Guignandon A , et al. Effects of long‐term microgravity exposure on cancellous and cortical weight‐bearing bones of cosmonauts. Lancet. 2000;355:1607‐1611. 10821365 10.1016/s0140-6736(00)02217-0

[45] Zerath E , Holy X , Roberts SG , et al. Spaceflight inhibits bone formation independent of corticosteroid status in growing rats. J Bone Miner Res. 2000;15:1310‐1320. 10893679 10.1359/jbmr.2000.15.7.1310

[46] Patterson‐Buckendahl P , Arnaud SB , Mechanic GL , et al. Fragility and composition of growing rat bone after one week in spaceflight. Am J Physiol. 1987;252:R240‐R246. 3812761 10.1152/ajpregu.1987.252.2.R240

[47] Jee WS , Wronski TJ , Morey ER , Kimmel DB . Effects of spaceflight on trabecular bone in rats. Am J Physiol. 1983;244:R310‐R314. 6829790 10.1152/ajpregu.1983.244.3.R310

[48] Landis WJ , Hodgens KJ , Block D , et al. Spaceflight effects on cultured embryonic chick bone cells. J Bone Miner Res. 2000;15:1099‐1112. 10841178 10.1359/jbmr.2000.15.6.1099

[49] Bikle DD . Integrins, insulin like growth factors, and the skeletal response to load. Osteoporos Int. 2008;19:1237‐1246. 18373051 10.1007/s00198-008-0597-z PMC9005159

[50] Tran T , Kolupaeva V , Basilico C . FGF inhibits the activity of the cyclin B1/CDK1 kinase to induce a transient G (2) arrest in RCS chondrocytes. Cell Cycle. 2010;9:4379‐4386. 21051949 10.4161/cc.9.21.13671 PMC3055189

[51] Song Y , Zhao C , Dong L , et al. Overexpression of cyclin B1 in human esophageal squamous cell carcinoma cells induces tumor cell invasive growth and metastasis. Carcinogenesis. 2008;29:307‐315. 18048386 10.1093/carcin/bgm269

[52] Ou Y , Ma L , Ma L , et al. Overexpression of cyclin B1 antagonizes chemotherapeutic‐induced apoptosis through PTEN/Akt pathway in human esophageal squamous cell carcinoma cells. Cancer Biol Ther. 2013;14:45‐55. 23114644 10.4161/cbt.22627 PMC3566051

[53] Shen X , Li Y , Sun G , et al. miR‐181c‐3p and ‐5p promotes high‐glucose‐induced dysfunction in human umbilical vein endothelial cells by regulating leukemia inhibitory factor. Int J Biol Macromol. 2018;115:509‐517. 29605252 10.1016/j.ijbiomac.2018.03.173

[54] Sun W , Min B , Du D , et al. miR‐181c protects CsA‐induced renal damage and fibrosis through inhibiting EMT. Febs Lett. 2017;591:3588‐3599. 28976551 10.1002/1873-3468.12872

[55] Yang G , Wu Y , Ye S . MiR‐181c restrains nitration stress of endothelial cells in diabetic db/db mice through inhibiting the expression of FoxO1. Biochem Biophys Res Commun. 2017;486:29‐35. 28223216 10.1016/j.bbrc.2017.02.083

