# Supplementary material for: miR‐181c‐5p mediates simulated microgravity‐induced impaired osteoblast proliferation by promoting cell cycle arrested in the G2 phase
Source: J Cell Mol Med. 2019 Feb 14;23(5):3302–16. doi: 10.1111/jcmm.14220 (PMC6484313; doi:10.1111/jcmm.14220)
Supplement: Supplementary file 1 [file JCMM-23-3302-s001.docx]

**Supporting figure 1**


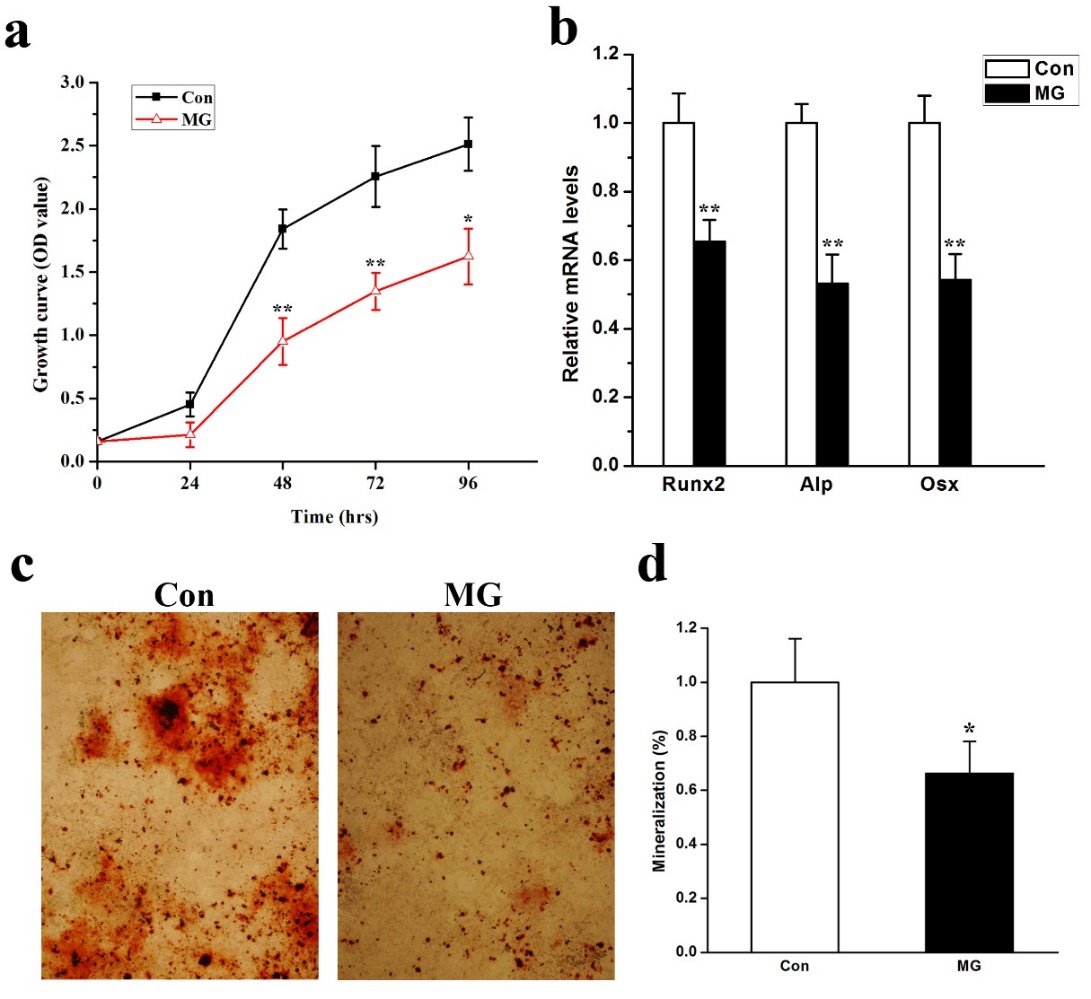


**Supporting figure 1.** Effects of simulated microgravity on biological characteristics of primary mouse osteoblasts. (a) Comparison of changes of cell growth between Con and MG group. The cells were seeded on 96-well plates at a density of 2,000 cells/well. Cell proliferation was evaluated by a WST-8 assay at 24-96 h (n=3). (b) Osteoblast differentiation was confirmed by qRT-PCR analysis of osteoblast marker genes (Runx2, Osx and ALP normalized to GAPDH) (n=3). (c) Effect of MG on the mineralization of osteoblasts. The figure was 40 × of the original section. (d) Quantitative colorimetric results (n=3). The results were expressed as the mean ± s.d. with two-tailed Student’s *t* test against control samples. ^*^*P* < 0.05 and ^**^*P* < 0.01, compared with stationary control.

**Supporting figure 2**


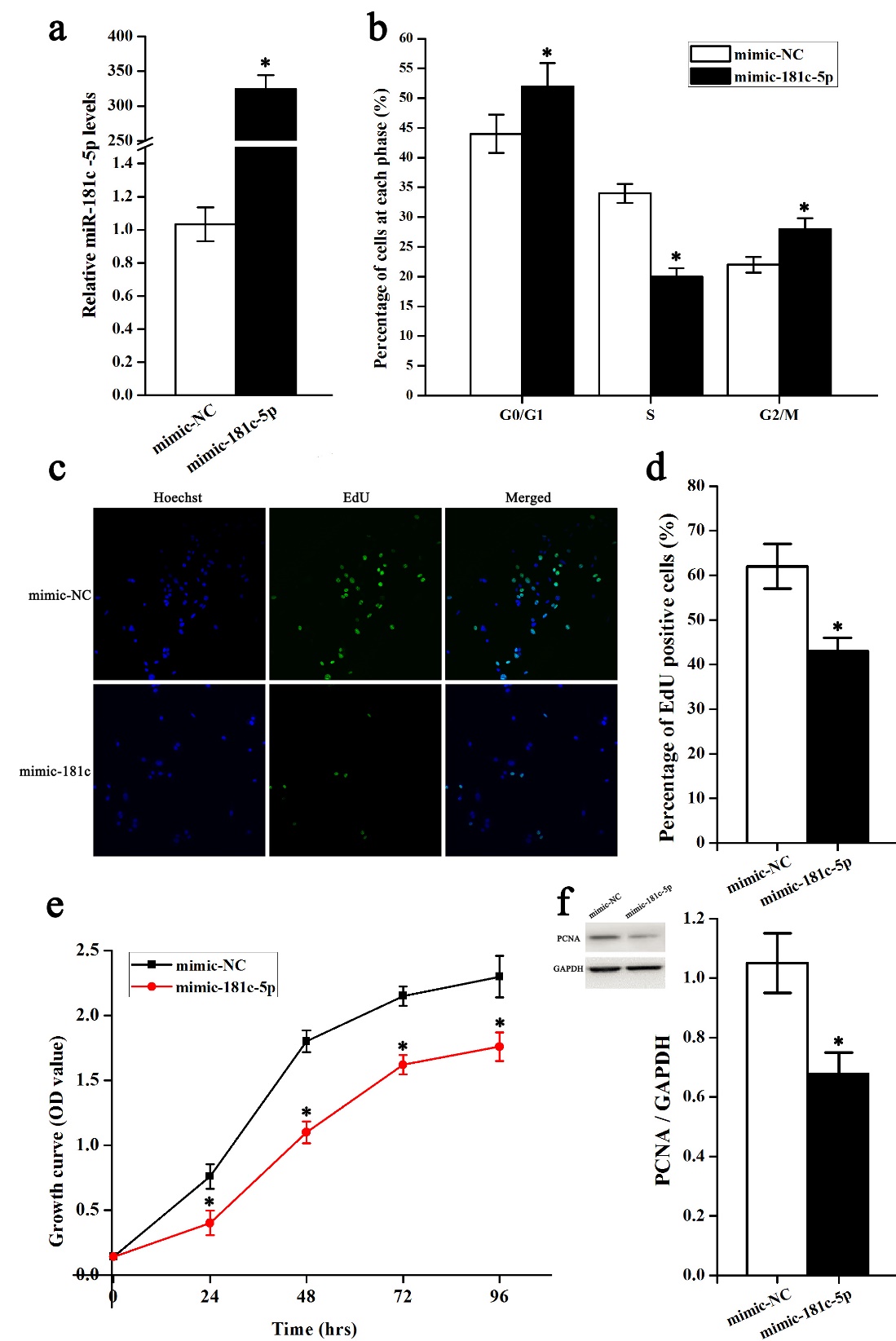


**Supporting figure 2.** The effect of miR-181c-5p on cell cycle and osteoblast proliferation. (a) qPCR analysis of the miR-181c-5p levels in osteoblasts to test the efficiency of mimic-181c-5p (n=3). (b) FCM analyses of osteoblasts transfected with mimic-181c-5p or mimic NC to examine the cell-cycle distribution. The percent of cells in each cycle stage was quantified and showed as histograms (n=3). (c) EdU labeling assays were analyzed using an inverted microscope linked to a confocal scanning unit. Proliferating osteoblasts were loaded with EdU. Osteoblasts were stained with the nucleic acid dye Hoechst (blue) and EdU (green). (d) Histogram of the percentage of EdU positive cells from different groups. The EdU incorporation rate was expressed as the ratio of EdU positive cells to total Hoechst positive cells (n=3). (e) Comparison of changes in cell growth among the different groups. Cells were seeded on 96-well plates at a density of 2,000 cells/well. Cell proliferation was evaluated by a CCK-8 assay at 24-96 h (n=3). (f) Western blot of PCNA expression in cells transfected with mimic-181c-5p or mimic NC. The total protein loaded per lane was 40 μg. Detection of GAPDH on the same blots was used to verify equal loading among the various lanes (n=3). The results were expressed as the mean ± s.d. with a one-way ANOVA with a SNK-q test. ^*^*P* < 0.05, compared with the stationary control.
